# Naturally Derived Silicone Surfactants Based on Saccharides and Cysteamine

**DOI:** 10.3390/molecules26164802

**Published:** 2021-08-08

**Authors:** Adrien Lusterio, Michael A. Brook

**Affiliations:** Department of Chemistry and Chemical Biology, McMaster University, 1280 Main St. W., Hamilton, ON L8S 4M1, Canada; lusteria@mcmaster.ca

**Keywords:** silicone surfactants, cysteamine thiol-ene, aminoalkylsilicones, saccharide hydrophiles, Piers–Rubinsztajn reaction

## Abstract

Silicone surfactants are widely used in many industries and mostly rely on poly(ethylene glycol) (PEG) as the hydrophile. This can be disadvantageous because commercial PEG examples vary significantly in polydispersity—constraining control over surface activity of the surfactant—and there are environmental concerns associated with PEG. Herein, we report a three-step synthetic method for the preparation of saccharide-silicone surfactants using the natural linker, cysteamine, and saccharide lactones. The Piers–Rubinsztajn plus thiol-ene plus amidation process is attractive for several reasons: if employed in the correct synthetic order, it allows for precise tailoring of both hydrophobe and hydrophile; it permits the ready utilization of natural hydrophiles cysteamine and saccharides in combination with silicones, which have significantly better environmental profiles than PEG; and the products exhibit interesting surface activities.

## 1. Introduction

**Figure 1:**
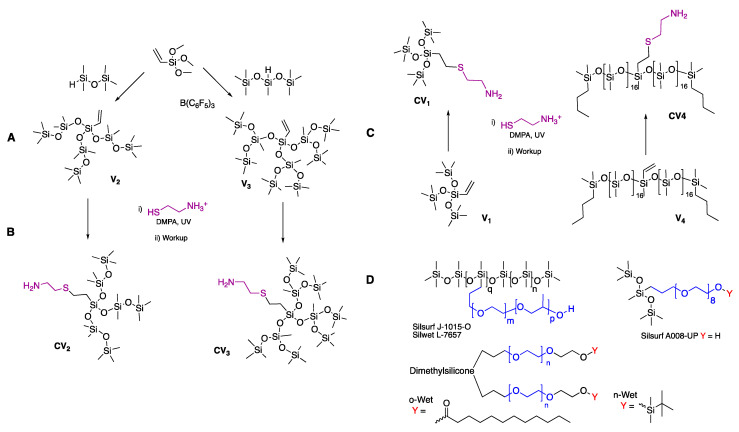
**A**: Preparation of **V2** and **V3** hydrophobes by the Piers–Rubinsztajn reaction. **B**,**C**: Conversion of these into aminoalkylsilicones **CV1–CV4** using cysteamine, as previously reported [1]. **D**: Structures of commercial silicone surfactants **Silwet L-7657**, **Silsurf J-1015-O**, **Silsurf A008-UP** (Siltech Corp.), and **n-Wet, o-Wet** (EnRoute Interfaces).

One important class of silicone polymers is surfactants. Traditional silicone surfactants ((Figure 1D) are generally produced by the hydrosilylation of allyl groups on poly(ethylene glycol) (PEG) with either silicone polymers or short chain oligomers. These compounds have found wide use in several industries including cosmetics, agriculture, coatings, and paints.

Silicone surfactants are almost always complex mixtures as a consequence of the commercial processes for the preparation of the silicone starting materials themselves, which do not lend themselves to precise polymers [2]. For example, while the process of hydrosilylation with allyl-PEG to make surfactants is very efficient, the silicone starting materials and the resulting products vary significantly in dispersity and degree of functionalization [3]. Unless very expensive precursors are used, the hydrophilic PEG components also contribute to surfactant complexity because the oligoethers are not monodisperse. 

Silicone surfactants undergo relatively rapid hydrolytic cleavage in the environment [4], as do linear silicones polymers, to give monomers that are more slowly converted by oxidation back to water, CO_2_, and sand [5]. There is increasing concern about the potential toxicity of PEG-based molecules, including the immunological response in humans to PEG-based materials and, additionally, in the environment [6]. It is of interest, therefore, to find alternative hydrophile sources of silicone surfactants that will have a better biological and environmental profile. Compounds based on alternative hydrophile structures may also possess different surface activities that will extend the utility of silicone surfactants. In addition, there is a desire to improve the sustainability of all chemicals, including silicone surfactants. 

There is a strong drive to increasingly use natural materials, including both monomers and polymers as constituents of ‘synthetic polymers’ [7]. Several researchers have examined the use of saccharides as the hydrophilic component in silicone surfactants due to their natural abundance, availability as single compounds, low cost, and friendly environmental profile; it is anticipated that saccharide-based moieties will undergo more facile degradation in the environment than their PEG analogues. 

A variety of synthetic strategies have been adopted for saccharide-silicone materials. Early processes utilized traditional saccharide chemistry [8,9,10] or hydrosilylation of allyl-amidosaccharides [11,12], both of which required alcohol protection/deprotection cycles. More recent approaches have taken advantage of protecting group-free syntheses that exploit reversible boronic ester linkages [13,14], bioprocessing with lipase to make sugar esters [15], and click chemistry [16,17], among others [17,18]. One of the simplest routes to saccharide-modified silicones utilizes ring-opening by commercial aminoalkylsilicones of sugar lactones that are generated by oxidation of the reducing end of sugars [19]. The reaction is normally rather facile to practice and is primarily limited by issues of miscibility [20,21]. Sugar-silicone fluids can exhibit a variety of interesting properties, including the ability to effectively dissipate energy [22,23]. 

Silicones have an excellent record for degradation in the environment to sand, water, and CO_2_, but there is a very high energy tax associated with their synthesis via silicon. We would like to exploit green(er) chemistry processes that are explicit, efficient, and that exploit renewable feedstocks [24]. Furthermore, we hypothesize that the use of a single surface active structure, rather than broad mixtures of surfactant molecules, should allow a more targeted approach in which a minimum amount of the ideal silicone surfactant can more effectively achieve the desired interfacial goal. That goal can only be achieved when precise libraries of compounds can be prepared that allow one, first, to establish structure–surface activity relationships and, then, design molecules with the desired characteristics.

As noted, linear silicone oligomers and polymers are almost always complex mixtures. By contrast, highly controlled silicone architectures required for precise surfactants, including those PEG-based silicone surfactants [3,25], result from the use of the precise, highly branched silicones that are available from the Piers–Rubinsztajn (PR) reaction (**V2**, **V3**, Figure 1A) [26,27]. The creation of sugar silicone surfactants from lactones requires an amidation that is not compatible with the PR reaction, as the complexation of amines with the required boron catalyst inhibits the process [28]. Clean methods for making precise aminosilicones are rather rare [21]. We recently reported that a thiol-ene reaction [29,30] between vinylsilicones and the natural amine cysteamine provides a solution to the synthesis of aminosilicones (**CV2**, **CV3**, Figure 1B) [1].

Herein, we report that the combination of these reactions in the correct synthetic order, PR–thiol-ene–amidation, permits the preparation of precise, highly branched sugar silicone surfactants. Libraries of green(er) explicit silicone architectures were prepared from natural saccharides and cysteamine. In addition to their syntheses, properties of the surfactants, including solubility, and emulsion stability, are described.

## 2. Materials and Methods

### 2.1. Materials

Gluconolactone, d-maltose monohydrate, lactobionic acid, cysteamine hydrochloride, and 2,2-dimethoxy-2-phenylacetophenone (DMPA) were purchased from Sigma Aldrich. Pentamethyldisiloxane (PMDS), bis(trimethylsiloxy)-methylsilane (BisH), vinyltrimethoxysilane, vinyltris(trimethylsiloxy)methylsilane (**V1**), and MCS-V212 (**V4**) were purchased from Gelest. Iodine was purchased from Anachemia, and potassium hydroxide and sodium bicarbonate were obtained from Caledon Laboratories. All compounds were used as received. Solvents methanol, ethanol, 2-propanol, hexane, dichloromethane, and toluene were also purchased from Caledon and dried through an activated alumina column as needed. Anhydrous methanol, deuterated chloroform (CDCl_3_), and deuterated DMSO-*d*_6_ were purchased from Sigma Aldrich and used as received. Neutral alumina was purchased from Fisher Chemical. A Celite S filter aid and Amberlite IR120, hydrogen form, the latter rinsed with distilled water before use, were purchased from Sigma Aldrich. Silsurf J1015-O and Silsurf A008-up were a gift from Siltech Corporation. Silwet L-7657 was purchased from Momentive. n-Wet and o-Wet were provided by EnRoute Interfaces, Inc. 

Compounds **V2**,**V3**, and **V1–V4 → CV1–CV4** have previously been reported [1]. We include, below, the preparation of **V2** and conversion to **CV2** to illustrate the simplicity of the process.

### 2.2. Methods

^1^H-NMR and ^13^C-NMR spectra were recorded with a Bruker AV-600 spectrometer (600 MHz) at room temperature using deuterated chloroform or deuterated DMSO as solvents and analyzed using Bruker Topspin software. Gas-chromatography/EI-mass spectrometry (GC/MS) was performed on a Waters Micromass GCT GC-EI/CI Mass Spectrometer (low resolution). Low resolution ESI spectra were performed using a Micromass/Waters Quattro Ultima ESI/APCI-LCMS Triple Quadruple Mass Spectrometer. High resolution ESI spectra were performed using an Agilent G1969 Time of Flight Mass Spectrometer. Infrared spectroscopy was performed using a Thermo Scientific Nicolet 6700 FT-IR spectrometer using a Smart iTX attenuated total reflectance (ATR) attachment. UV-photochemistry was performed using a high intensity UV Lamp by Analytik Jena (UVP-B-100AP) at 365 nm and 100 watts. Rotary evaporation was performed using a BUCHI R-210 Rotavapor at 50 Torr. Short-path vacuum distillation was performed using kugelrohr B-585 from BUCHI. Lyophilization was performed using a STELLAR^®^ Laboratory Freeze Dryer by Millrock Technology.

### 2.3. Synthesis of Vinyl Pecursors

#### 2.3.1. Synthesis of Vinyltris(pentamethyldisiloxanyl)silane (**V2**) 

A 100 mL oven-dried, round-bottomed flask was purged under nitrogen for 10 min (Figure 1). Vinyltrimethoxysilane (1.56 g, 10.5 mmol), dry toluene (2 mL), and B(C_6_F_5_)_3_ catalyst (0.67 mL of 10 mg mL^−1^ solution, 0.016 mmol) were added, and the mixture was stirred under nitrogen. The desired hydrosilane, for example, pentamethyldisiloxane (PMDS) for **V2** (6.25 g, 42.1 mmol) was added dropwise, using a needle and syringe, in a slight stoichiometric excess (4.0 equivalent for **V2** based on the 3 alkoxy groups); subsequent drops were added only once bubbling had ceased for a total addition time of ~20 min. Neutral alumina (~0.1 g) was added to the flask after stirring for 1 h to quench the B(C_6_F_5_)_3_ catalyst. The solution was gravity filtered and the alumina was rinsed with hexanes, followed by rotary evaporation of the combined liquid fractions to remove solvent. Rotary evaporation was sufficient to remove residual pentamethyldisiloxane in the case of **V2**. Yields **V2** 4.81 g (84%).

**V2**: ^1^H-NMR (CDCl_3_, 600 MHz): δ 5.98–5.87 (m, 3H), 0.09 (m, 45H) ppm. GCMS-EI [M-CH_3_]^+^ *m*/*z* at 529 (13), 367 (17), 341 (23), 221 (55), 41 (45), 73 (55). 

**V3**: ^1^H-NMR (CDCl_3_, 600 MHz): δ 5.98–5.86 (m, 3H), 0.09 (m, 63H) ppm. GCMS-EI [M-CH_3_]^+^ *m*/*z* at 751 (7), 401 (15), 355 (15), 295 (52), 221 (100), 147 (82), 73 (96). 

#### 2.3.2. Aminofunctionalization of Vinyl-Silicones with Cysteamine Hydrochloride V→CV

The desired vinyl-functional T-unit silicone (Table 1, Figure 1) **V1**–**V3**, and cysteamine hydrochloride were added to an oven-dried 250 mL round-bottomed flask and dissolved in a 50/50 *v*/*v* solution of 2-propanol/ethanol, which led to a transparent cysteamine hydrochloride/silicone solution. The photoinitiator DMPA (5 mol%), dissolved in 2-propanol (2 mL), was added to the flask. The flasks were covered with aluminum foil, only exposing one side to the 365 nm UV lamp (100 watts, 1.27 W cm^−2^). The solution was irradiated for 1 h; the resulting solution was slightly yellow and transparent. The workup included neutralizing HCl by adding NaHCO_3_ (3 equivalent to cysteamine∙HCl) to the solution and stirring for 1 h, followed by filtration through Celite. The recovered alcohol solution was concentrated under vacuum. Hexanes (~50 mL) were added to precipitate any excess cysteamine. Filtration through Celite and rotary evaporation of the filtrate afforded the aminofunctional silicone products **CV1**–**CV4** as viscous oils that required additional drying under a stream of N_2_ for 24 h. The products were characterized using ^1^H-NMR; the disappearance of vinyl groups and the appearance of methylene signals corresponding to cysteamine derivatives confirmed the assigned structures. Mass spectrometric data supported structural assignments. An analogous process was used to modify the commercial monovinylsilicone MCS-V212 (**V4**).

**CV1**: ^1^H-NMR (CDCl_3_, 600 MHz): δ 2.82 (t, 2H, *J* = 6.4 Hz), 2.57 (t, 2H, *J* = 6.3 Hz), 2.49–2.46 (m, 2H), 1.97 (s, 2H, broad, NH_2_ protons), 0.76–0.73 (m, 2H), 0.04 (s, 27H) ppm. ^13^C-NMR (CDCl_3_, 150 MHz): δ 41.0, 35.8, 26.6, 15.8, 1.8 ppm. ESI [M+H]^+^ at *m*/*z* 400.2, [M+Na]^+^ at *m*/*z* 422.

**CV2**: ^1^H-NMR (CDCl_3_, 600 MHz): δ 2.84 (t, 2H, *J* = 6.2 Hz), 2.60–2.54 (m, 4H, overlapping), 1.51 (s, 2H, NH_2_ protons), 0.88–0.83 (m, 2H), 0.07–0.03 (m, 45H). ^13^C-NMR (CDCl_3_, 150 MHz): δ 41.2, 36.3, 26.5, 15.7, 1.9, 1.2 ppm. GCMS EI [M-CH_3_]^+^ at *m*/*z* 606.2.

**CV3**: ^1^H-NMR (CDCl_3_, 600 MHz): δ 2.86 (t, 2H, *J* = 6.4 Hz), 2.62–2.59 (m, 4H, overlapping), 1.75 (s, 2H, broad, NH_2_ protons), 0.91–0.88 (m, 2H), 0.09–0.04 (m, 63H) ppm. ^13^C-NMR (CDCl_3_, 150 MHz): δ 41.3, 36.4, 26.4, 15.7, 1.9, −1.9. ESI [M+H]^+^ at *m*/*z* 844.3. 

**MCS-V212 (V4)** Starting Material: ^1^H-NMR (CDCl_3_, 600 MHz): δ 6.01 (dd, 1H, *J* = 14.9, 19.7 Hz), 5.93 (dd, 1H, *J* = 14.9, 4.6 Hz), 5.80 (dd, 1H, *J* = 19.8, 4.6 Hz), 1.33–1.30 (m, 8H, overlapping), 0.89 (t, 6H, *J* = 6.9 Hz), 0.54 (t, 4H, *J* = 9.2 Hz), 0.09–0.05 (m, 204H) ppm. 

**CV4**: ^1^H-NMR (CDCl_3_, 600 MHz): δ 2.86 (t, 2H, *J* = 6.1 Hz), 2.62 (t, 2H, *J* = 6.2 Hz), 2.57 (t, 2H, *J* = 8.9 Hz), 1.49 (s, 2H, broad, NH_2_ protons), 1.33–1.31 (m, 8H, overlapping), 0.89–0.86 (m, 8H, overlapping), 0.53 (t, 4H, *J* = 9 Hz), 0.08–0.04 (m, 204H, overlapping), ppm. ^13^C-NMR (CDCl_3_, 150 MHz): 0.3, 1.2, 14.0, 18.1, 18.7, 25.6, 26.4, 26.5, 36.3, 41.3 ppm.

### 2.4. Synthesis of Saccharide Lactones 

Maltonolactone was prepared following the literature procedures [31]. To a solution of d-maltose (1.8 g, 5 mmol) dissolved in H_2_O (20 mL) was added iodine (2.54 g, 10 mmol) in H_2_O (100 mL) and potassium hydroxide (2.23 g, 40 mmol) in H_2_O (20 mL). The solution was stirred at room temperature for 4 h, followed by passing through a cation exchange column packed with Amberlite IR-120 resin that was rinsed with distilled water prior to use. Silver carbonate (5.5 g, 20 mmol) was added to the recovered solution to precipitate iodide, followed by filtration. The solution was passed through the cation exchange column a second time, which was then regenerated with 1M HCl. Conversion to the lactone from d-maltonic acid was achieved by lyophilizing for 24 h to yield d-maltonolactone (1.47 g, 82% yield) as a white powder. FTIR v(C=O) 1734 cm^−1^. ^13^C-NMR (DMSO-*d*_6_, 150 MHz): C=O 172.6 ppm. 

Lactobionolactone was prepared from commercially available lactobionic acid (Sigma Aldrich, St. Louis, MO, USA) following literature procedures [32,33]. Lactobionic acid (3.0 g, 8.37 mmol) was dissolved in anhydrous methanol (30 mL) by stirring at 50 °C over 12 h. A small amount of trifluoroacetic acid (~5 μL) was added, followed by vacuum distillation. The process was repeated twice, yielding lactobionolactone (2.49 g, 87% yield) as a white powder. FTIR v(C=O) 1734 cm^−1^. ^13^C-NMR (DMSO-*d*_6_, 150 MHz): (C=O) 173.9 ppm. 

### 2.5. General Procedure for Sugar Silicones via Amide Formation 

Into a 25 mL round-bottomed flask was added 1 equivalent of the chosen aminofunctional silicone and 1 equivalent of the chosen sugar lactone (Table 2). Methanol and/or ethanol was added, depending on the molar mass of the silicone—higher molar mass silicones utilized both—and the reaction was stirred at room temperature for 24–48 h. Solvents were removed using rotary evaporation and the resulting products were dissolved in dichloromethane. Unreacted sugar lactone, if observed via FTIR, was then removed by gravity filtration, and the filtrate was concentrated under vacuum to obtain the products in good yield. In general, completed reactions with 1:1 ratios of amine:sugar resulted in no observable residual lactone peaks in the FTIR, and required no additional purification. The products were characterized with FTIR, ^1^H-NMR, ^13^C-NMR, and mass spectroscopy.

**GluCV1**: ^1^H-NMR (CDCl_3_, 600 MHz): δ 0.09–0.11 (m, 27H), 0.79 (t, 2H, *J* = 8.7 Hz), 2.54 (t, 2H, *J* = 8.7 Hz,), 2.67 (t, 2H, *J* = 6.3 Hz), 3.40–5.24 (m, 13H, broad and overlapping CH_2_NHCO and glucose signals) ppm. ^13^C-NMR (CDCl_3_, 150 MHz): δ 1.9, 15.7, 26.9, 31.3, 38.6, 63.8, 70.5, 71.9, 73.1, 74.2, 173.2 ppm. FT-IR: 1648 cm^−1^ (ν C=O)). HRMS (ES positive mode): *m*/*z* [M+Na]^+^ calc. = 600.1941; found = 600.1945.

**MalCV1**: ^1^H-NMR (CDCl_3_, 600 MHz): δ 0.09–0.11 (m, 27H), 0.78–0.91 (m, 2H), 2.51–2.54 (m, 2H), 2.63–2.65 (m, 2H), 2.81–5.38 (m, 23H, broad and overlapping CH_2_NHCO and maltose signals) ppm. ^13^C-NMR (CDCl_3_, 150 MHz): δ 1.84, 15.8, 26.7, 34.8, 38.8, 40.8, 61.5, 63.1, 70.0 - 73.6 several overlapped peaks), 82.5, 101.0, 173.3 ppm. FT-IR: 1648 cm^−1^ (ν C=O)). HRMS (ES positive mode): *m*/*z* [M+H]^+^ calc. = 740.2650; found = 740.2652. 

**LacCV1**: ^1^H-NMR (CDCl_3_, 600 MHz): δ 0.08–0.11 (m, 27H), 0.78 (m, 2H), 2.53–2.75 (m, 4H, overlapping), 3.03–5.87 (m, 22H, broad and overlapping CH_2_NHCO and lactose signals) ppm. ^13^C-NMR (CDCl_3_, 150 MHz): δ 1.9, 15.6, 26.8, 31.2, 38.8, 39.8, 61.9, 62.9, 69.3–75.5 (several overlapped peaks), 82.2, 104.3, 173.3 ppm. FT-IR: 1650 cm^−1^ (ν C=O)). HRMS (ES positive mode): *m*/*z* [M+Na]^+^ calc. = 762.2469; found = 762.2473. 

**GluCV2**: ^1^H-NMR (CDCl_3_, 600 MHz): δ 0.06–0.09 (m, 45H), 0.84–0.91 (m, 2H), 2.58–2.60 (m, 2H), 2.66–2.68 (t, 2H, *J* = 6.5 Hz), 3.41 - 5.27 (m, 13H, broad and overlapping CH_2_NHCO and glucose signals) ppm. ^13^C-NMR (CDCl_3_, 150 MHz): δ 1.3, 2.0, 15.6, 26.7, 31.5, 38.4, 63.9, 70.7, 72.0, 73.2, 74.3, 172.9 ppm. FT-IR: 1649 cm^−1^ (ν C=O)). HRMS (ES positive mode): *m*/*z* [M+Na]^+^ calc. = 822.2505; found = 822.2517. 

**MalCV2**: ^1^H-NMR (CDCl_3_, 600 MHz): δ 0.05–0.09 (m, 45H), 0.82–0.92 (m, 2H), 2.50–2.71 (m, 4H), 2.81–5.49 (m, 23H, broad and overlapping CH_2_NHCO and maltose signals) ppm. ^13^C-NMR (CDCl_3_, 150 MHz): δ 0.9-2.0, 15.2, 26.3, 29.8, 31.3, 39.2, 62.8–73.9 (several overlapped peaks), 173.8 ppm. FT-IR: 1647 cm^−1^ (ν C=O)). HRMS (ES positive mode): *m*/*z* [M+Na]^+^ calc. = 984.3033; found = 984.3028.

**LacCV2**: ^1^H-NMR (CDCl_3_, 600 MHz): δ 0.06–0.09 (m, 45H), 0.85–0.91 (m, 2H), 2.53–2.68 (m, 4H), 2.85–5.49 (m, 23H, broad and overlapping CH_2_NHCO and lactose signals) ppm. ^13^C-NMR (CDCl_3_, 150 MHz): δ 1.3, 2.0, 15.8, 26.6, 31.4, 38.8, 61.8, 62.7, 69.3–75.6 (several overlapped peaks), 104.2, 173.3 ppm. FT-IR: 1650 cm^−1^ (ν C=O)). HRMS (ES positive mode): *m*/*z* [M+Na]^+^ calc. = 984.3033; found = 984.3035.

**GluCV3**: ^1^H-NMR (CDCl_3_, 600 MHz): δ 0.05–0.10 (m, 63H), 0.87–0.90 (m, 2H), 2.61–2.68 (m, 4H, overlapping), 3.44–5.10 (m, 13H, broad and overlapping CH_2_NHCO and glucose signals) ppm. ^13^C-NMR (CDCl_3_, 150 MHz): δ −1.9, 1.9, 15.5, 26.6, 31.6, 38.3, 64.0, 70.8, 72.0, 73.2, 74.2,172.6 ppm. FT-IR: 1652 cm^−1^ (ν C=O)). HRMS (ES positive mode): *m*/*z* [M+Na]^+^ calc. = 1044.3068, found = 1044.3073.

**MalCV3**: ^1^H-NMR (CDCl_3_, 600 MHz): δ 0.05–0.11 (m, 63H), 0.87–0.90 (m, 2H), 2.61–2.72 (m, 4H, overlapping), 2.80–5.50 (m, 23H, broad and overlapping CH_2_NHCO and maltose signals) ppm. ^13^C-NMR (CDCl_3_, 150 MHz): δ −1.9, 1.9, 15.5, 26.7, 31.6, 38.8, 62.2–74.7 (several overlapped peaks), 82.2, 100.9, 172.9 ppm. FT-IR: 1648 cm^−1^ (ν C=O). HRMS (ES positive mode): *m*/*z* [M+Na]^+^ calc. = 1206.3597; found = 1206.3583.

**LacCV3**: ^1^H-NMR (CDCl_3_, 600 MHz): δ 0.03–0.13 (m, 63H), 0.85–0.91 (m, 2H), 2.51–2.73 (m, 4H, overlapping), 2.83–5.40 (m, 23H, broad and overlapping CH_2_NHCO and lactose signals) ppm. ^13^C-NMR (CDCl_3_, 150 MHz): δ −1.9, 1.9, 15.4, 26.7, 31.6, 39.4, 62.0, 63.0, 69.4–75.4 (several overlapped peaks), 82.44, 104.49, 173.06 ppm. FT-IR: 1650 cm^−1^ (ν C=O)). HRMS (ES positive mode): *m*/*z* [M+Na]^+^ calc. = 1206.3597; found = 1206.3608.

**GluCV4**: ^1^H-NMR (CDCl_3_, 600 MHz): δ 0.03–0.08 (m, 204H), 0.53 (t, 4H, *J* = 6.6 Hz), 0.88 (t, 8H, *J* = 7.02), 1.27–1.34 (m, 8H, overlapping signals), 2.57–2.60 (m, 2H), 2.66–2.70 (m, 2H), 2.81–4.92 (m, 13H, broad and overlapping CH_2_NHCO and glucose signals) ppm. ^13^C-NMR (CDCl_3_, 150 MHz): δ 0.3, 1.2, 13.9, 18.1, 18.5, 25.6, 26.5, 31.5, 38.4, 63.9, 70.6, 71.9, 73.1, 74.1, 172.9 ppm. FT-IR: 1650 cm^−1^ (ν C=O).

**MalCV4**: ^1^H-NMR (CDCl_3_, 600 MHz): δ 0.04–0.08 (m, 204H), 0.54 (t, 4H, *J* = 6.2 Hz), 0.88 (t, 8H, *J* = 6.8 Hz), 1.28–1.34 (m, 8H, overlapping signals), 2.55–2.59 (m, 2H), 2.62–2.69 (m, 2H), 2.85–2.89 (m, 1H), 3.06–5.62 (m, 22H, broad and overlapping maltose signals) ppm. ^13^C-NMR (75% CDCl_3_, 25% DMSO-*d*_6_, 150 MHz): δ −0.9–0.06 (overlapping signals), 12.7, 13.2, 16.7, 17.34, 25.0, 25.1, 27.4, 28.4, 38.0, 38.8 (DMSO), 55.91, 59.38–72.75 (several peaks), 80.66, 98.72, 174.63 ppm. FT-IR: 1650 cm^−1^ (ν C=O).

**LacCV4**: ^1^H-NMR (CDCl_3_, 600 MHz): δ 0.04–0.07 (m, 204H), 0.53 (t, 4H, *J* = 6.2 Hz), 0.88 (t, 8H, *J* = 6.2 Hz), 1.27–1.34 (m, 8H, overlapping signals), 2.55–2.60 (m, 2H), 2.64–2.70 (m, 2H), 2.88–5.55 (m, 23H, broad and overlapping lactose signals) ppm. ^13^C-NMR (CDCl_3_, 150 MHz): δ 0.3, 1.2, 13.9, 18.1, 18.4, 25.6, 26.5, 31.4, 38.8, 62.2, 63.2, 64.7–75.4 (several overlapped peaks), 82.32, 104.42, 173.23 ppm. FT-IR: 1650 cm^−1^ (ν C=O).

### 2.6. Surface Tension Measurements

Surface tension was measured by the pendant drop method using a Krüss DSA 10 drop shape analysis system equipped with an environmental chamber; measurements were made at 23.0 ± 0.5 °C and performed using solutions prepared in deionized water at various concentrations. Drops were formed with 1.8 ± 0.1 mm o.d. blunt syringe needle tips, and drop volumes varied from 12 to 20 µL. The measurement started after the pendant drop had stabilized for 20 s. For every sample, 3–5 points were measured at 5 s intervals. Average values and standard deviations are reported (Figure S7, SI). A critical micelle concentration could only be measured for the disaccharide **LacCV1**, the most hydrophilic of the compounds that dispersed the best in water. Note that a comparison between mono vs. disaccharides was only made in **CV1–CV4** compounds made from glucose and lactobionic acid, respectively.

### 2.7. Emulsion Stability Tests

Experiments to assess emulsion stability in 1:1 water:D_5_ emulsions were made (49.5 wt% H_2_O, 49.5 wt% D_5_, and 1 wt% of the surfactant). The surfactant was measured into a vial and then blended with the appropriate amount of the silicone (D_5_) phase, followed by mixing. Then, the water phase was added. To ensure adequate mixing of both phases, a Dremel 3000 Variable Speed Rotary Tool with steel brush was used to mix each vial at 5000 rpm for 10 s. The appearance of the mixtures was noted at various time intervals to determine when the emulsion became unstable. Synthesized compounds were compared with commercial surfactants (Table S1, Supplementary Materials).

## 3. Results and Discussion

Traditional synthetic methods for silicone—linear or branched—surfactants often lead to loss of silicone structural integrity due to acid/base catalyzed depolymerization of silicone chains [34]. The PR reaction [2] allows for the facile assembly of silicones with highly controlled and diverse 3D architectures without depolymerization/metathesis. Three-fold symmetry vinylsiloxanes **V2** and **V3** were prepared from commercial vinyltrimethoxysilane (Figure 1A); such branched structures are not found in commercial silicone polymers or surfactants. These architectures are highly tunable; further variation of the bulk (hepta- or deca-siloxanes, **V2** vs. **V3**) and degree of branching (one-, two-, or three-fold symmetry) is possible by simply changing the number of alkoxy groups on the vinyl starting material and structure of the hydrosilane [2]. The vinyl functionality was retained after the reaction; B(C_6_F_5_)_3_ catalyzed hydrosilylation was not observed at the low catalyst loadings used (0.05 mol%) [28].

While, in principle, it should be possible to create tris derivatives (tris = ~Si(OSiMe_3_)_3_) using Me_3_SiH and the PR reaction, the volatility and flammability of Me_3_SiH precluded these experiments and, therefore, vinylSi(OSiMe_3_)_3_ **V1** was purchased. A commercial monovinylsilicone polymer **V4** contributed a larger, more complex hydrophobic moiety to the library.

Relative reactivity dictated that the thiol-ene reaction had to follow the PR process, since the boron catalyst is inactivated by the presence of good Lewis bases such as amines [28]. Thiol-ene reactions were performed using cysteamine hydrochloride with compounds **V1**–**V3** (three-fold symmetry) and **V4** (two-fold symmetry); molar masses of the starting materials ranged from 323 g mol^−1^ for **V1** to ~2700 g mol^−1^ for **V4**, the latter estimated by ^1^H-NMR. As previously reported [1], the two reaction partners—hydrophobic silicones and water soluble cysteamine hydrochloride—were conveniently able to dissolve in mixtures of ethanol and isopropanol. A slight stoichiometric excess of the thiol was required, in each case, for complete conversion. Irradiation at 365 nm for 1 h in the presence of 2,2-dimethoxy-2-phenylacetophenone (DMPA) as photoinitiator led to the product aminoalkylsilicones **CV1**–**CV4** in over 80% yields (Figure 1B,C). Note that excess cysteamine was removed simply by precipitation in hexane and filtration. The thiyl radical formed in the presence of DMPA underwent an anti-Markovnikov addition to the vinyl group exclusively [1,35], as shown by ^1^H-NMR (**V1** → **CV1**, Supplementary Materials).

Several other advantages are associated with this approach to aminoalkylsilicones: the thiol-ene process only required mild workup procedures, including removal of the excess cysteamine through precipitation and filtration and neutralization of the cysteamine hydrochloride with NaHCO_3_; the reaction was conducted in benign alcohol solvents [36]; and, most important, amine protecting groups such as *tert*-butoxycarbonyl were not required [37]. The ingredients and methods are both facile and better follow the tenets of green chemistry than traditional processes [24]; we continue to look for better alternatives to the current photocatalyst.

**Figure 2:**
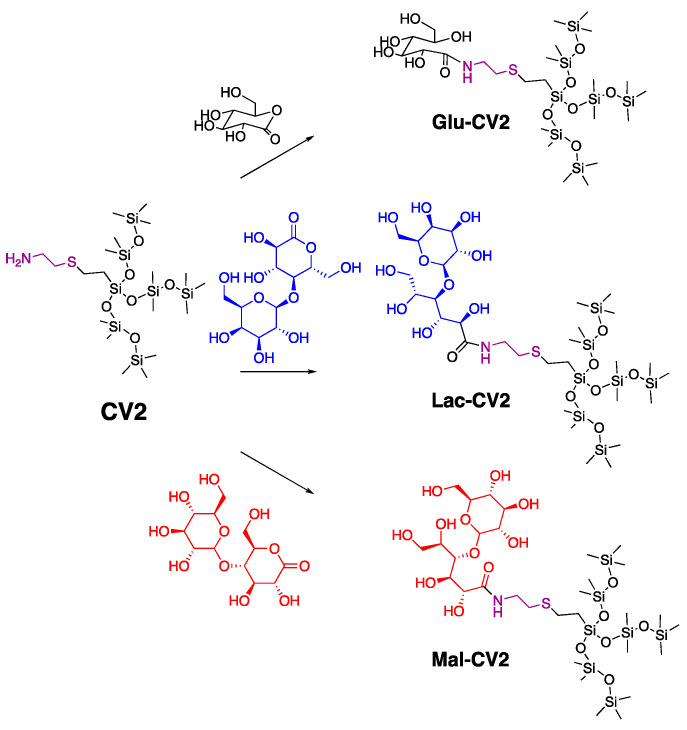
Synthesis of selected sugar silicones.

**CV1**–**CV4** were each modified with three different sugar lactones to make a library of surfactants—a monosaccharide based on glucose and disaccharide lactones [20] based on maltose and lactobionic acid, respectively—to form sugar silicones (Figure 2, shown for the reaction of the **CV2** with the three different sugars). These reactions proceeded under mild conditions, simply by stirring at room temperature for 24–48 h, and generated no by-products [19]. If excess saccharide was accidently added, it was readily removed by precipitation in non-polar solvents and filtration. Conversion to the amide was clear from the FT-IR, which showed the disappearance of the lactone-ester stretch in the range 1720–1750 cm^−1^ and appearance of an amide signal at ~1650 cm^−1^. Several observable changes arising from the formation of the sugar aminoalkylsilicone products from their precursor amino silicones were also observed with ^1^H-NMR and ^13^C-NMR and IR (**CV1**, Supplementary Materials). The products were isolated in good yields of 78–93% (Table 2). The products **GluCV**, **MalCV**, and **LacCV** ranged from viscous oils to solids, depending on both the molar mass and structure of the hydrophobic/hydrophilic constituents (Table 3).

We focus, in this report, on small, readily available, reducing sugars, as oxidation to the lactone at the reducing end allows ready formation of sugar-silicone surfactants. It should be possible to broaden the range of saccharides used to make surfactants, including polysaccharides [19]; many other natural hydrophiles possess, or are easily modified to include, lactones. Saccharide hydrophiles avoid any environmental concerns that may be associated with polyethers and create materials with narrow ranges of properties; most oligoethers of ethylene or propylene glycol are relatively polydisperse [3,25].

The solubility properties of **GluCV**, **MalCV**, and **LacCV** were tested in water, isopropanol, toluene, and, in low molar mass, silicone oil (D_5_, (Me_2_SiO)_5_). In a 1% by weight solution, all compounds were soluble in D_5_, 2-propanol, and toluene (Table 3).

In water, the solubility/dispersibility of the compounds (1 wt%) were less affected by the sugar hydrophile than the size of the silicone hydrophobe (Table 3). The products derived from larger hydrophobes, **SugarCV3** and **SugarCV4**, were neither soluble nor dispersible in water; even after sonication, a clear solution with solid ‘chunks’ throughout was observed. However, dispersions of 1% surfactant in water could be made of **SugarCV1** and **SugarCV2**. The stability of the dispersions depended on both the sugar and silicone component; a dispersion of **LacCV1** was stable after 5 days, while the analogous mixture of **LacCV2** physically separated out over that time period (Figure S6, Supplementary Materials).

Silicones may both be hydrophobic and lipophobic (insoluble in normal hydrocarbons). Three-dimensional HLB calculations [38,41], an enhanced system designed to reflect the differences between silicone and normal surfactants, conveniently reflect these behaviors [38,41]. While HLB measurements compare hydro/lipophilicity, the 3D HLB system considers oil, water, and silicone components. Prediction of surfactant behavior can be done using the calculated (x,y) coordinates in the scaled triangle system where each constituent represents a vertex of the triangle. The 3D HLB coordinates are calculated using the percentage of the water soluble hydrophile divided by five for the x coordinate, and the percentage of the oil soluble component divided by five for the y coordinate. **Mal/LacCV1** and **Mal/LacCV2**, with 46.2% and 36.1% hydrophile-by-weight content, respectively, were able to disperse in water, while surfactants derived from glucose, **GluCV1** and **GluCV2**, which have 31.2% and 22.5% hydrophile-by-weight (3D HLB values of (6.23, 3.57) and (4.50, 2.58), respectively, Table 3), dispersed much less well than those made with disaccharides.

Attempts to use surface tension measurements to determine CMC of the sugar silicone surfactants were unsuccessful, except for **LacCV1**, the most hydrophilic and water soluble of the compounds prepared (3D HLB value of 9.24, 2.79), which had a CMC of 1.1mM (Figure S7, Supplementary Materials), comparable to analogous PEG-based surfactants [25]. The remaining compounds, notably those with HLB (hydrophile component of 3D HLB) within the range of 1.22–6.23, had too much silicone to be useful in water, but were deemed to be suitable stabilizers for water-in-oil emulsions based on the 3D HLB parameters; all of the surfactants were soluble in D_5_ (W/O emulsions, Table 3. Note that the oil being considered in this paper is silicone rather than hydrocarbon oil).

The ability of these surfactants (1 wt%) to stabilize water-in-silicone oil emulsions (50:50 water:D_5_ W/O emulsions)[42,43] was compared with several commercial PEG-based emulsifiers and wetting agents. After initial mixing, the compounds were allowed to rest without further agitation; changes in turbidity and phase separation were used to judge surfactant efficacy and a baseline comparison of emulsion stability [39]. Glucose compounds with large, branched silicone hydrophobes **GluCV2** and **GluCV3** (3D HLBs of (4.50, 2.58) and (3.52, 2.02)) remained stable for longer time periods than their disaccharide counterparts **MalCV2** and **MalCV3** (3D HLBs of (7.22, 2.18) and (5.78, 1.74)). However, with smaller silicone hydrophobes, the reverse trend was observed; the maltose compound **MalCV1** (3D HLB of 9.24, 2.79) remained stable longer (20 min) than the glucose derivative **GluCV1** (3D HLB of 6.23, 3.57). The surfactants that led to the most stable emulsions were **GluCV4** and **MalCV4**, which remained stable for ≥2 h, but none survived for 10.5 h (Table S1, Supplementary Materials). These data follow expected outcomes; bigger and more viscous silicone anchors are more effective at reinforcing the O/W interface.

At the same (low) surfactant weight% (1%), many of the synthesized compounds led to comparable or greater emulsion stability than several structurally different commercial silicone surfactants (Table S1, Supplementary Materials). For example, **MalCV1** (3D HLB of 9.24, 2.79) and **GluCV1** (3D HLB of 6.23, 3.57) had comparable stability to commercial compounds **Silsurf J-1015-O** and **n-Wet**, which are block copolymers (Figure 1D). The most stabilizing **GluCV4** and **MalCV4** were comparable to commercial compounds **o-Wet** and **Silsurf A008-UP**, which have block copolymer and superwetter structures, respectively.

The nature of the silicone structural motifs affects the surface behavior of silicone surfactants [44,45,46]. Small silicone surfactants (up to tetrasiloxanes) respond differently to oil/water interfaces, based solely on the 3D structures of the silicone head groups. For example, only branched trisiloxane surfactants ((Me_3_SiO)_2_SiMe-linker-hydrophile) exhibit superwetting activity, a property exploited in agricultural adjuvants [47]. This is ascribed to highly efficient packing at the interface [45]. **GluCV4** and **MalCV4**, which both have a two-fold branched structure, were similarly effective at stabilizing an O/W interface, while the three-fold surfactants made from **CV1–CV3** fared less well.

The power of the 3D HLB [38] tool shows up in these data. The scale was originally based on linear silicone hydrophobes and linear polyether hydrophiles, which are hydrogen-bonding, but which do not contain OH groups. An examination of the structural motifs of sugar silicone surfactants and commercial surfactants (Figure 1, Figure 2, Table S1, Supplementary Materials) show few common features, yet the 3D HLB mostly predicts their behavior. There were some subtle variations in behavior, for example, effective dispersion follows the order **GluCV2** > **MalCV2**, but **MalCV1** > **GluCV1**; this can be ascribed to the subtle playoff of mono/disaccharide hydrophile and small/medium 3D silicone hydrophobe. Current research is focused on preparing surfactant libraries exploiting the process so that more detailed structure–surface activity relationships can be teased out.

Green and sustainable chemistry are evolving paradigms in both academic and industrial chemistry. As noted above, there exist some concerns about PEG ethers, which are derived from petroleum. The replacements used here—cysteamine and a variety of sugars—are renewable, biodegradable, and readily available. The photocatalyzed thiol-ene process currently requires an aromatic photosensitizer; the catalyzed process is efficient. We are currently examining greener, natural photosensitizers for the reaction. The second, amide forming reaction has no by-products, does not need catalysts, and is efficient under mild conditions [20]. Thus, the synthetic process constitutes a greener strategy to silicone surfactants. These surfactants are readily tunable both in their hydrophilic and hydrophobic domains. The process above focused on three-fold, branched silicone hydrophobes, particularly to stabilize water-in-silicone oil emulsions. However, the same PR process can be used to make lower order hydrophobes of various lengths, including rake type polymers (e.g., analogous to Silsurf J-1015-O, Figure 1). The range of available saccharides is even wider, including those with lower and higher densities of hydrogen-bonding entities, and linear and branched structures. 

## 4. Conclusions

The combination of thiol-ene reactions between cysteamine and vinylsilicones and the ring-opening reaction of the amine product with sugar lactones leads to a high yield of sugar silicone surfactants. This approach avoids the need for functional group protection, and takes advantage of mild, radical conditions that avoid metal catalysts and that are relatively insensitive to oxygen in the atmosphere. The hydrophilic constituents are renewable and, therefore, have benefits when considering sustainability. The control of the architectures and the silicone-saccharide interface is made possible through the natural organic linker cysteamine. Although only three sugars were examined, any saccharidic material with a reducing end that can be oxidized to a lactone should similarly participate in the process. The materials were mostly insoluble in water due to the low surface energy of the silicone constituents, but the surface activity is readily modified by judicious choice of both silicone and saccharidic components. For the samples examined, the high silicone content lends their use, in particular, to the formulation of water-in-silicone oil emulsions. 

## Figures and Tables

**Table 1:** Cysteamine-Silicone Formulations.

Vinyl-Silicone Starting Material	Mass of VinylsiliCone g (mmol)	Mass of Cysteamine∙HCl g (mmol)	Solvent (mL)	Mass of DMPA g (mmol)	Product: Isolated Yield g (%)
**V1** ^a^	3.50 (10.84)	1.50 (13.20)	EtOH (30)	0.16 (0.65)	**CV1**: 3.46 (80)
**V2**	2.0 (3.67)	0.54 (4.77)	1:1 IPA/EtOH (15)	0.06 (0.24)	**CV2**: 1.92 (84)
**V3**	3.14 (4.09)	0.61 (5.32)	1:1 IPA/EtOH (20)	0.07 (0.27)	**CV3**: 2.87 (83)
**V4**	4.0 (1.48)	0.59 (5.19)	1:1 IPA/EtOH (30)	0.07 (0.27)	**CV4**: 3.67 (89)

^a^ Vinyltris(trimethylsiloxy)silane (Sigma Aldrich). Nomenclature: products from **V** are **CV**. Formulations follow those previously reported for these compounds [1].

**Table 2:** Formulas for Sugar Silicone Formation.

Starting Aminosilicone	Sugar Lactone	Solvent (mL)	Product: Isolated Yield in g (%)
**CV1** (0.33 g, 0.83 mmol) (0.30 g, 0.75 mmol) (0.30 g, 0.75 mmol)	Gluconolactone (0.147 g, 0.83 mmol)	MeOH (10)	**GluCV1** 0.40 (84)
	Maltonolactone (0.25 g, 0.75 mmol)	MeOH (10)	**MalCV1** 0.47 (85)
	Lactobionolactone (0.25 g, 0.75 mmol)	MeOH (10)	**LacCV1** 0.46 (82)
**CV2** (0.22 g, 0.35 mmol)	Gluconolactone (0.06 g, 0.35 mmol)	MeOH (9)	**GluCV2** 0.26 (93**)**
	Maltonolactone (0.12 g, 0.35 mmol)	MeOH (9)	**MalCV2** 0.26 (78)
	Lactobionolactone (0.12 g, 0.35 mmol)	MeOH (9)	**LacCV2** 0.28 (82)
**CV3** (0.19 g, 0.22 mmol)	Gluconolactone (0.04 g, 0.22 mmol)	MeOH (8)	**GluCV3** 0.20 (91)
	Maltonolactone (0.076 g, 0.22 mmol)	MeOH (8)	**MalCV3** 0.21 (82)
	Lactobionolactone (0.076 g, 0.22 mmol)	MeOH (8)	**LacCV3** 0.20 (79)
**CV4** (0.30 g, 0.11 mmol)	Gluconolactone (0.019 g, 0.11 mmol)	MeOH (4), EtOH (4)	**GluCV4** 0.28 (87)
	Maltonolactone (0.038 g, 0.11 mmol)	MeOH (4), EtOH (4)	**MalCV4** 0.29 (86)
	Lactobionolactone (0.037 g, 0.11 mmol)	MeOH (4), EtOH (4)	**LacCV4** 0.28 (83)

**Table 3:** Behavior of branched silicone sugars (1 wt%) in various solutions.

Compound	Product Description	3D HLB	Solubility in H_2_O ^[a]^	Emulsion Type(s) by 3D HLB ^[b]^
**GluCV1**	Waxy solid	6.23, 3.57	Sparingly dispersible	W/O,
**MalCV1**	Glassy solid	9.24, 2.79	Dispersible	O/W[c]
**LacCV1**	Glassy solid	9.24, 2.79	Dispersible	O/W[c]
**GluCV2**	Waxy solid	4.50, 2.58	Sparingly dispersible	W/O
**MalCV2**	Glassy solid	7.22, 2.18	Dispersible	W/O[d]
**LacCV2**	Glassy solid	7.22, 2.18	Dispersible	W/Od]
**GluCV3**	Waxy solid	3.52, 2.02	Insoluble	W/O
**MalCV3**	Waxy solid	5.78, 1.74	Insoluble	W/O
**LacCV3**	Waxy solid	5.78, 1.74	Insoluble	W/O
**GluCV4**	Viscous oil	1.22, 0.70	Insoluble	W/O
**MalCV4**	Viscous oil	2.19, 0.66	Insoluble	W/O
**LacCV4**	Viscous oil	2.19, 0.66	Insoluble	W/O

^[a]^ All compounds were soluble in D_5_, toluene, and isopropanol. ^[b]^ Indicates emulsion type based on 3D HLB coordinates [38,39,40] O refers to silicone oil. ^[c]^ Si/W refers to silicone oil/water emulsion. Despite high HLB values, these compounds exhibit strong solubility in D_5_ compared to water. ^[d]^ Compounds with borderline 3D HLB values are classified as W/O due to their high affinity for silicone.

## Data Availability

Data are contained within the article or supplementary material.

## Supplementary Materials

The following are available online. ^1^H NMR spectra of cysteamine products. IR and NMR spectra of sugar silicones **Glu-CV1**, CMC plot of **LacCV1**, Table of emulsion stabilities.

## Sample Availability

Samples of the compounds are not available from the authors.
