# Supplementary material for: Naturally Derived Silicone Surfactants Based on Saccharides and Cysteamine
Source: Molecules. 2021 Aug 8;26(16):4802. doi: 10.3390/molecules26164802 (PMC8399498; doi:10.3390/molecules26164802)
Supplement: Supplementary file 1 [file molecules-26-04802-s001.zip › molecules-1318947-supplementary.pdf]

Preparation of Highly Branched Sugar Silicone Surfactants using Cysteamine

Adrien Lusterio and Michael A. Brook*
Department of Chemistry and Chemical Biology
1280 Main St. W., Hamilton ON Canada L8S 4M1.

Supporting Information

Amino-functionalization with Cysteamine

The feasibility of thiol-ene radical addition reactions of cysteamine hydrochloride was demonstrated with various vinyl-functional silicone hydrophobes. ^{1}H-NMR shows the conversion of vinyl peaks to new methylene peaks corresponding to the cysteamine adduct, as example, shown for products **CV1** (Figure S1) and **CV4** (Figure S2). The results are also supported with ^{13}C-NMR (shown in Figure S5 in comparison to the amidified-glucose product). Similar and successful reactions in making **CV2**-**CV4** followed, indicating the robustness of the process.

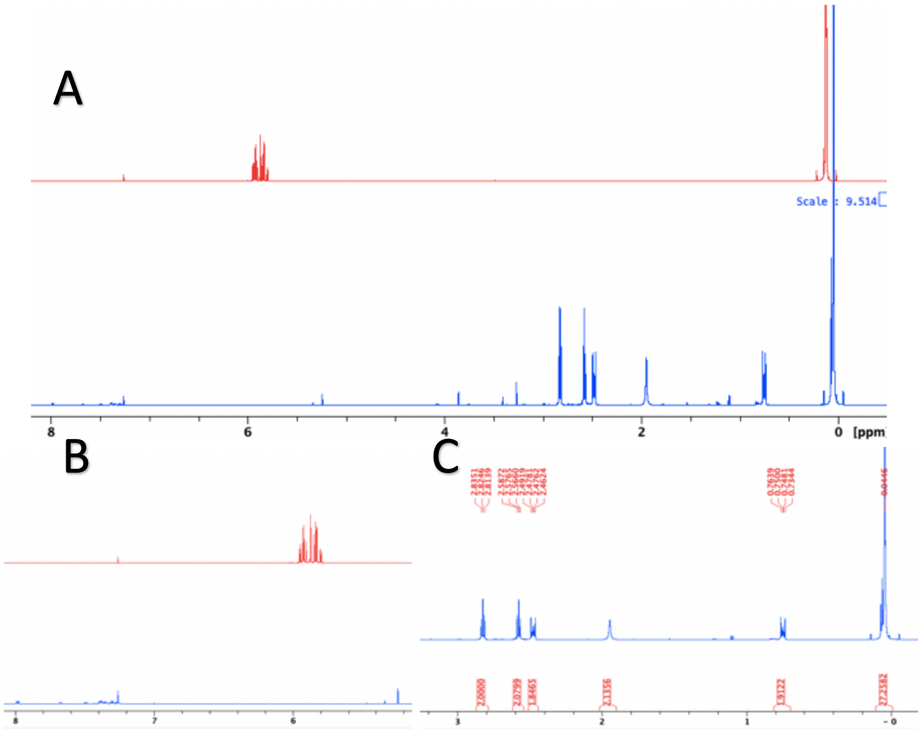

Figure S1: ^{1}H-NMR of the starting material **V1** (red) and the aminoalkylsilicone product, **CV1** (blue). A: Full spectrum, B: expanded region showing consumption of vinyl protons (note some minute residual 2,2-dimethoxy-2-phenylacetophenone signals), C: expanded region of the four new methylene signals consistent with the anti-Markovnikov addition of cysteamine.

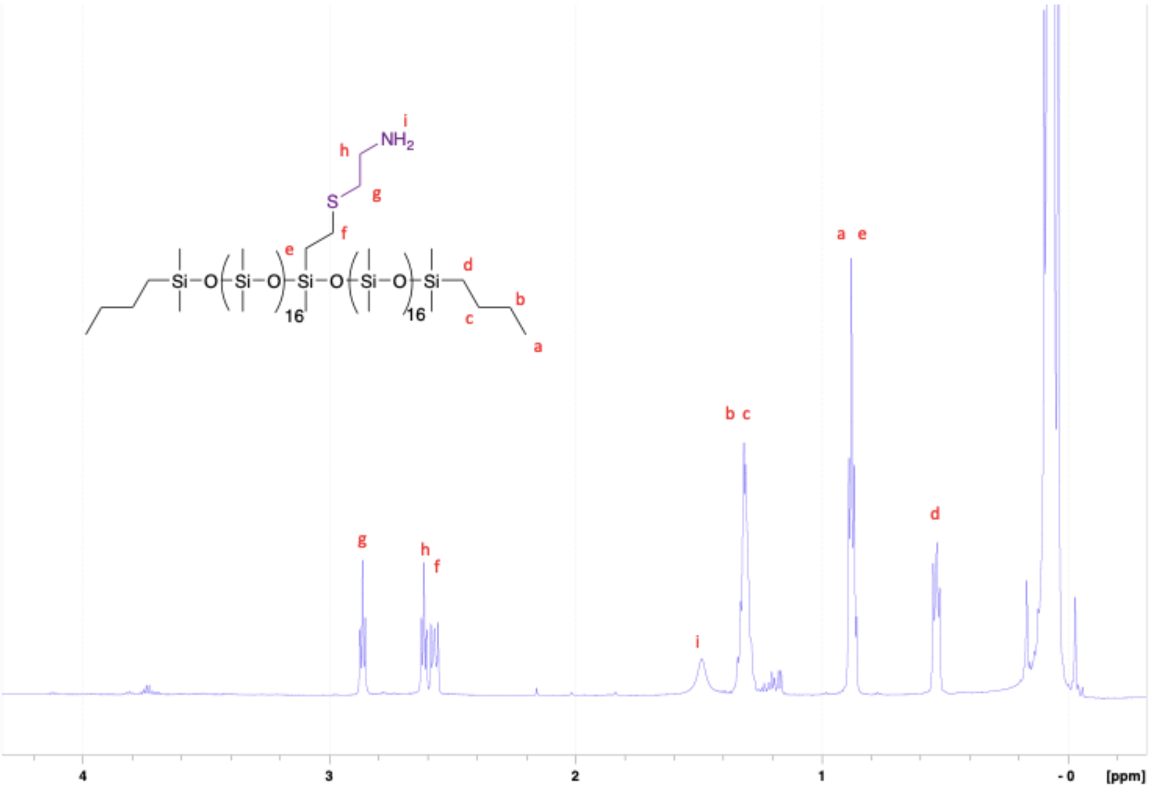

Figure S2: ^{1}H-NMR of compound CV4, with peak assignments consistent with the functionalization of cysteamine.

Lactone ring-opening with amino-alkyl-silicones

The feasibility of the amidification reactions of the synthesized monofunctional-amino-alkyl-silicones with the sugar lactones was initially followed by FTIR to verify the formation of an amide bond consistent with the amine attacking to open the lactone ring. This was verified by the complete disappearance of the lactone C=O signal (~1730 cm^{-1}) and the appearance of a new amide C=O signal at ~1650 cm^{-1} (seen for all sugar-cysteamine-silicones). For example, see the changes in FTIR as amino-alkyl-silicone **CV1** is converted with gluconolactone and lactobionolactone, respectively, to give **GluCV1** or **LacCV1** (Figure S3). This process was also followed with ^{1}H-NMR (for **CV1** → **GluCV1**, see Figure S4). Analogous outcomes can be seen in the ^{13}C-NMR spectra (Figure S5).

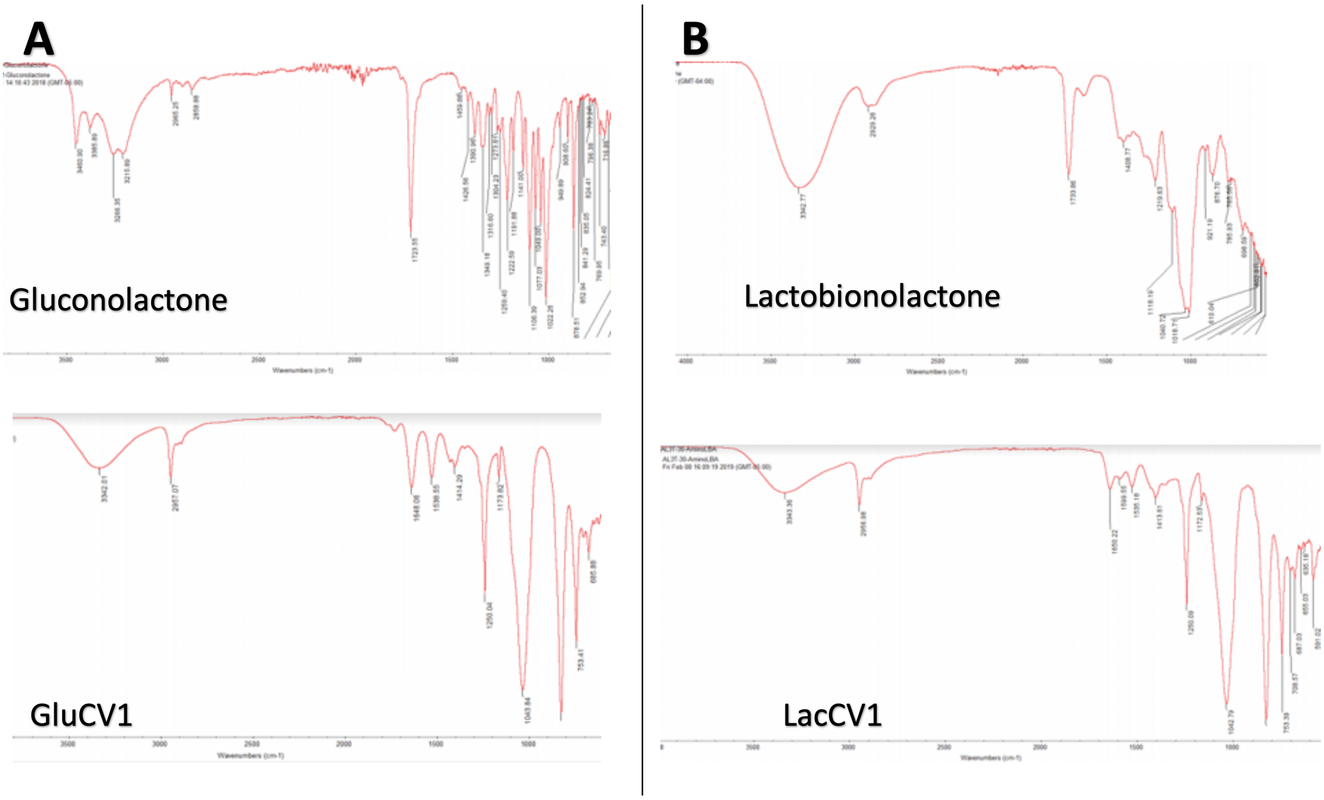

Figure S3: FTIR of lactone sugars and subsequent conversion to amides with amino-alkyl-silicone **CV1** for A) gluconolactone to give **GluCV1** or B) lactobionolactone to give **LacCV1**; the disappearance of the ester carbonyl stretches above 1720 cm^{-1} and the appearance of amide carbonyl stretches around 1650 cm^{-1} can be seen.

Changes in the NMR of select compounds after sugar modification

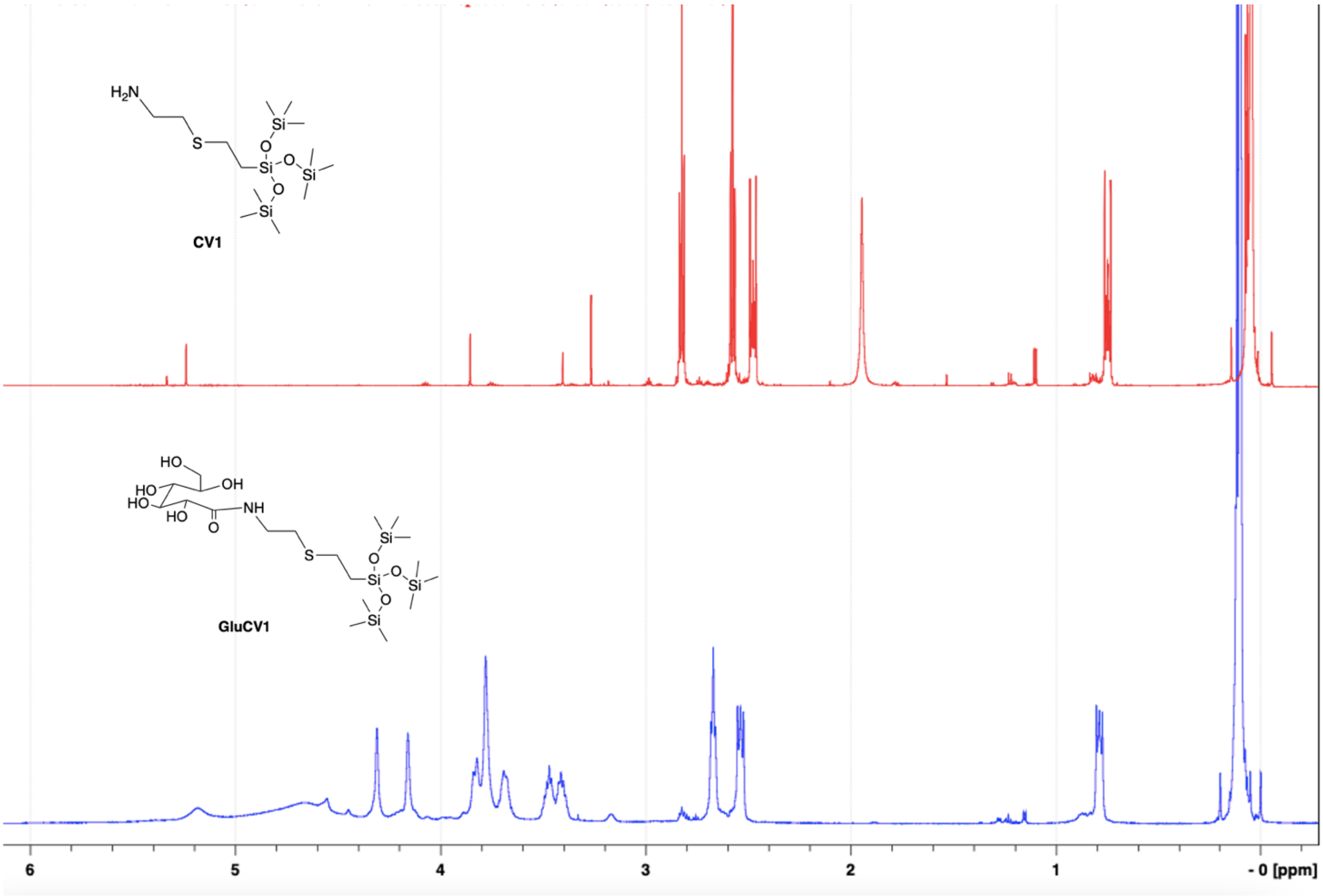

Figure S4: ^{1}H-NMR data of **CV1** (top) and gluconolactone-modified product **GluCV1** (bottom). There are shifts of the methylene signals adjacent to nitrogen once the amide is formed from the amine. In addition, there is disappearance of the broad NH_2 protons of CV1 at ~1.9 ppm, and corresponding appearance of glucose signals between ~2.80 - 5.4 ppm of GluCV1.

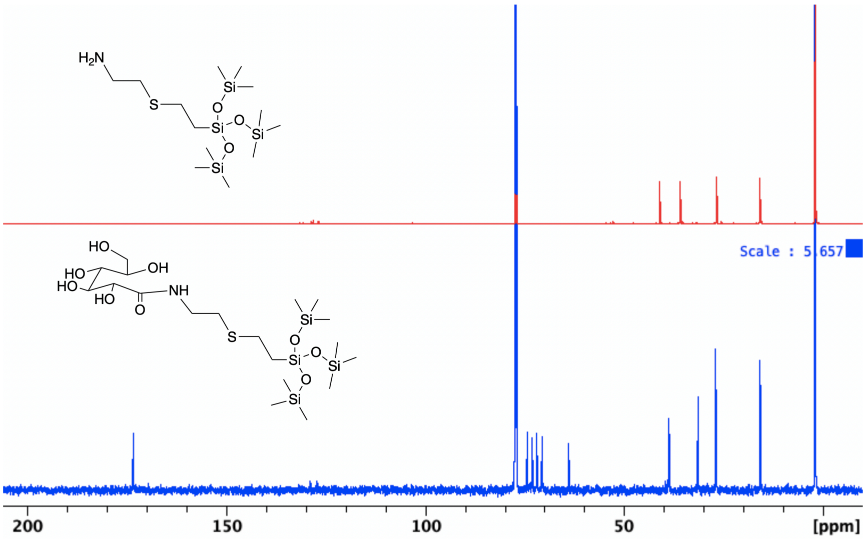

Figure S5: ^{13}C-NMR of **CV1** (top) and **GluCV1** (bottom). Notable shifts of the CH_2CH_2NHCO carbons are observed in the region of 20-40 ppm, consistent with the new amide environment. Notable signals of **GluCV1** also appear; the carbonyl at ~173 ppm and several glucose signals from ~63-77 ppm.

Stability of selected 1 wt % dispersions in water after 5 days

Dispersions in water of **LacCV1** and **LacCV2** were monitored over 5 days by allowing the solutions to sit without disruption. After 5 days, the disaccharide with the smaller silicone **CV1** remained as a dispersion. However, the disaccharide modified by only a slightly larger silicone, **CV2**, changed from a dispersion into insoluble chunks that floated in suspension after 5 days (Figure S6).

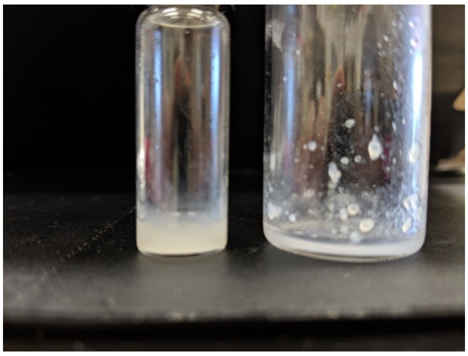

Figure S6: Images after 5 days of 1 wt% dispersion of **LacCV1** (left) and **LacCV2** (right) in water. The former shows a dispersion that did not show any differences from its initial preparation. The latter, a compound with a larger silicone/hydrophile ratio, rapidly converted to suspended macroscopic particles.

Critical Micelle Concentration of compound LacCV1

A critical micelle concentration could only be measured for the disaccharide **LacCV1** (Figure S7), the most hydrophilic of the compounds that dispersed the best in water. Note that a comparison between mono vs disaccharides was only made in **CV1-CV4** compounds made from glucose and lactobionic acid, respectively

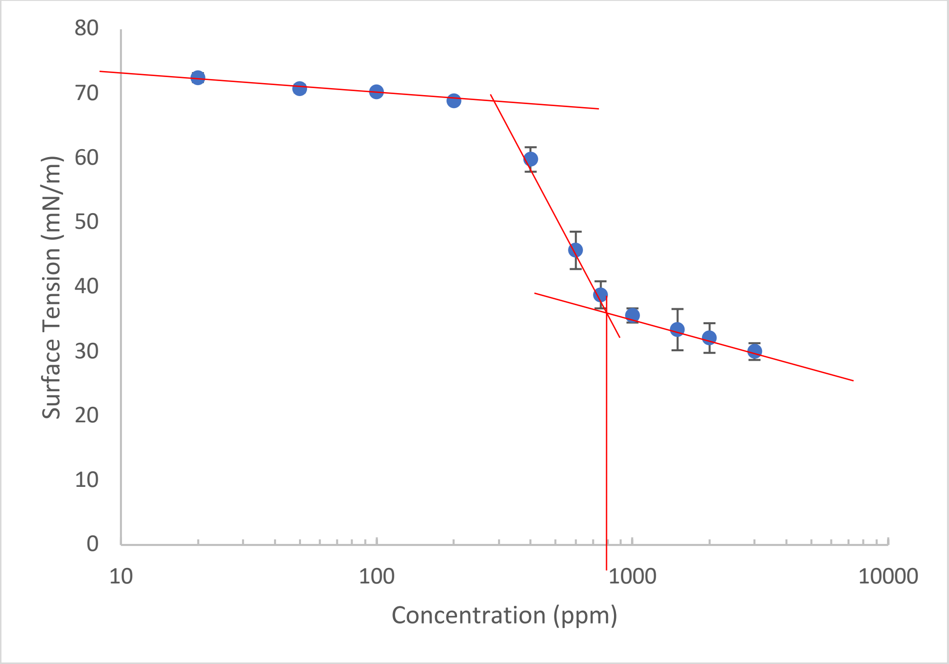

Figure S7: Surface Tension data for **LacCV1**.

Calculation for CMC:
800 ppm (graph) = 800 mg/L
0.8g/L / 740.12g/mol * 1000mM/M = 1.1 mM

Emulsion Stability Tests

Emulsion stability (time until visible separation), comprised of 1 wt% surfactant in 49.5/49.5 wt% solutions of D_5/Water, was evaluated for synthesized and commercial surfactants by noting appearance at various time intervals (Table S1).

Table S1. Emulsion stability (time until visible separation) of synthesized and commercial surfactants at 1wt% (of total emulsion) surfactant in 49.5/49.5 wt% solutions of D_5/Water.

Time after mixing (min)	Emulsifier GluCV1	GluCV2	GluCV3	GluCV4	MalCV1	MalCV2	MalCV3	MalCV4
0	Stable	Stable	Stable	Stable	Stable	Stable	Stable	Stable
10	Starting to break	Stable	Stable	Stable	Stable	Starting to break	Starting to break	Stable
20	Broken	Stable	Stable	Stable	Starting to break	Broken	Broken	Stable
30	Broken	Stable	Stable	Stable	Broken	Broken	Broken	Stable
60	Broken	Broken	Starting to break	Stable	Broken	Broken	Broken	Stable
120	Broken	Broken	Broken	Stable	Broken	Broken	Broken	Stable
630	Broken	Broken	Broken	Broken	Broken	Broken	Broken	Broken
1440	Broken	Broken	Broken	Broken	Broken	Broken	Broken	Broken

Commercial Surfactants

Time after mixing (min)	Emulsifier Silwet L-7657	Silsurf J-1015-O	Silsurf A008-UP	n-Wet	o-Wet
0	Stable	Stable	Stable	Stable	Stable
10	Starting to break	Starting to break	Stable	Stable	Stable
20	Broken	Broken	Stable	Stable	Stable
30	Broken	Broken	Stable	Starting to break	Stable
60	Broken	Broken	Stable	Broken	Stable
120	Broken	Broken	Stable	Broken	Stable
630	Broken	Broken	Broken	Broken	Broken
1440	Broken	Broken	Broken	Broken	Broken
